# Reforming solid tumor treatment: the emerging potential of smaller format antibody-drug conjugate

**DOI:** 10.1093/abt/tbae005

**Published:** 2024-02-16

**Authors:** Xiaojie Ma, Mingkai Wang, Tianlei Ying, Yanling Wu

**Affiliations:** MOE/NHC/CAMS Key Laboratory of Medical Molecular Virology, Shanghai Frontiers Science Center of Pathogenic Microorganisms and Infection, Shanghai Institute of Infectious Disease and Biosecurity, School of Basic Medical Sciences, Shanghai Medical College, Fudan University, Shanghai 200032, China; MOE/NHC/CAMS Key Laboratory of Medical Molecular Virology, Shanghai Frontiers Science Center of Pathogenic Microorganisms and Infection, Shanghai Institute of Infectious Disease and Biosecurity, School of Basic Medical Sciences, Shanghai Medical College, Fudan University, Shanghai 200032, China; MOE/NHC/CAMS Key Laboratory of Medical Molecular Virology, Shanghai Frontiers Science Center of Pathogenic Microorganisms and Infection, Shanghai Institute of Infectious Disease and Biosecurity, School of Basic Medical Sciences, Shanghai Medical College, Fudan University, Shanghai 200032, China; Shanghai Engineering Research Center for Synthetic Immunology, Fudan University, Shanghai 200032, China; MOE/NHC/CAMS Key Laboratory of Medical Molecular Virology, Shanghai Frontiers Science Center of Pathogenic Microorganisms and Infection, Shanghai Institute of Infectious Disease and Biosecurity, School of Basic Medical Sciences, Shanghai Medical College, Fudan University, Shanghai 200032, China; Shanghai Engineering Research Center for Synthetic Immunology, Fudan University, Shanghai 200032, China

**Keywords:** antibody-drug conjugates, solid tumors, smaller antibody formats, single-domain antibody, tumor penetration

## Abstract

In recent years, substantial therapeutic efficacy of antibody-drug conjugates (ADCs) has been validated through approvals of 16 ADCs for the treatment of malignant tumors. However, realization of the maximum clinical use of ADCs requires surmounting extant challenges, mainly the limitations in tumor penetration capabilities when targeting solid tumors. To resolve the hurdle of suboptimal tumor penetration, miniaturized antibody fragments with engineered formats have been harnessed for ADC assembly. By virtue of their reduced molecular sizes, antibody fragment-drug conjugates hold considerable promise for efficacious delivery of cytotoxic agents, thus conferring superior therapeutic outcomes. This review will focus on current advancements in novel ADC development utilizing smaller antibody formats from ~6 to 80 kDa, with particular emphasis on single-domain antibodies, which have been widely applied in novel ADC design. Additionally, strategies to optimize clinical translation are discussed, including half-life extension, acceleration of internalization, and reduction of immunogenic potential.

## Teaser

Statement of Significance: The research on ADCs is advancing toward an innovative transitional phase. Small antibody fragments or other structurally derived scaffolds from natural proteins have been employed for the development of ADCs, aiming to overcome the limitations of traditional ADCs and enhance their therapeutic effects in solid tumor treatment.

## INTRODUCTION

Antibody-drug conjugates (ADCs) have been hailed as ‘biological missile’ for their ability to selectively deliver cytotoxic payloads to tumor cells, thereby potentially improving the therapeutic index of small molecule cytotoxic agents [1]. The first ADC approved by US Food and Drug Administration (FDA) in 2000, Mylotarg® (gemtuzumab ozogamicin) was used for treatment of adult acute myeloid leukemia (AML) [1, 2]. As of August 2023, a total of 16 ADCs have been approved globally for hematological malignancies and solid tumors, and over 100 ADC candidates are undergoing clinical trials [3, 4].

ADC consists of a tumor targeting monoclonal antibodies conjugated to a cytotoxic payload via sophisticatedly designed chemical linker, enabling the ability of precise targeting and potent effectiveness simultaneously [4]. ADCs predominantly employ conventional immunoglobulin G (IgG) antibodies, with high affinity that enables effective internalization while preserving plasma half-life [5]. Fc receptors can also induce robust effector functions such as antibody-dependent cell-mediated cytotoxicity (ADCC), antibody-dependent phagocytosis (ADCP), and complement-dependent cytotoxicity (CDC) [6]. The cytotoxic payload serves as active component that induces cytotoxicity upon internalization of ADCs into cancer cells. The efficacy and adverse effects of ADCs are determined by cytotoxic payloads [7]. Currently, potent tubulin inhibitors, DNA damaging agents, and immunomodulators are predominantly employed as cytotoxic payloads for ADCs, including monomethyl auristatin E (MMAE), monomethyl auristatin F (MMAF), mertansine (DM1), SN-38, pyrrolobenzodiazepine dimers (PBD), doxorubicin (DOX), and toll-like receptor activators [4, 8]. Notably, site-specific conjugation has emerged as a prominent technique for next-generation ADCs, including smaller format ADCs [9–12] (Table 1).

**Table 1:** A list of small format drug-conjugates in order of size with examples of related targets and payloads

Format	Size (kDa)	Example targets	Cancer	DAR	Example payload	Reference
IgG	~150	CD30	Lymphoma	4	MMAE	[1]
SIP Minibody VHH-Fc	~80	Fibronectin	TME	2	DM1	[42]
Diabody	~80	MSLN	Pancreatic cancer	3.6	MMAE	[62]
Fab	~55–60	CD30	Lymphoma	2	MMAF	[41]
ScFv	~50	HER2	Breast cancer	1	PBD	[23]
	~25	GD2	Neuroblastoma	1	MMAE	[35]
Nb	~12.5–15	5 T4	Pancreatic cancer	1	SN38	[58]
VNAR	~12.5–25	BCR	Lymphoma	1	MMAE	[52]
Adnectin	~10	EGFR	SCLC	1	MMAF	[65]
DARPin	~14–17	EpCAM	SCLC	2	MMAE	[68]
Affibody	~6–7	HER3	Breast cancer	1	DM1	[74]
Pentarin	~3–5	SSTR 2	SCLC	1	DM1	[77]
Bicycle	~1.5–2	EphA2	Prostatic cancer	1	MMAE	[79]

Despite substantial interest in ADC therapies, high failure rates have been observed clinically [13]. Efficacious tumor accumulation is imperative for antitumor activity of ADCs, especially against solid tumors, which is contingent on both tumor penetration/retention and antibody pharmacokinetics [14]. First, the delivery of ADCs from intravenous (IV) injection site to tumors poses challenges for specific distribution and stability [15]. Second, large sizes of conventional ADCs (MW > 150 kDa) restrict drug accumulation in solid tumor [16]. Last but importantly, most ADCs exhibit nonspecific release and accumulation in healthy cells, conferring unintended toxicity. Furthermore, Fc interactions may mediate off-target ADC internalization and toxicity in immune cells [16, 17].

Considering the issues mentioned above, small antibody fragments or other structurally scaffolds derived from natural proteins have been used for ADCs development, such as single-chain variable fragments (scFvs), antigen-binding fragments (Fabs), functional heavy-chain variable domain, and various antibody-like scaffolds [18, 19]. In this review, we categorize drug conjugations involving small antibody formats into four main groups: antibody fragments derived from conventional IgG (Fab, scFv, Bivalent antibody-derived fragments); single-domain antibodies (sdAbs); antibody-like scaffolds, peptides and molecules. Additionally, we also discuss their development as targeted therapies for solid tumors, as well as strategies to improve their limitations for clinical applications.

## BASED ON ANTIBODY FRAGMENTS

### Antibody binding fragment (Fab)

Among various antibody formats, Fab fragments contain the constant and variable domains of Igs without the Fc domains [20]. Approaches for ADC conjugation are also applicable to generating Fab-conjugated drugs, which are especially suitable for dialkylated drug conjugation owing to the single solvent-accessible interchain disulfide bond [21, 22]. Previous studies have demonstrated that compared with ADCs, Fab-drug conjugates possess certain advantageous characteristics.

A classical Fab-drug conjugate is trastuzumab-MMAE with DAR of 1, exhibiting potent cytotoxicity (IC_50_ 200–500 pM) *in vitro*. Compared with trastuzumab ADC, MMAE conjugation to Fab via bis-alkylation showed superior homogeneity and stability. This Fab conjugate exhibited potent tumor growth inhibition in a mouse BT-474 breast cancer model, albeit requiring high dose of 20 mg/kg every other day over 25 days [23]. SG3710, another trastuzumab Fab conjugate, utilized an ultra-potent PBD payload and demonstrated pM-range IC_50_. This ADC rebridged the hinge cysteines at position 220 to produce a hydrophilic ADC with DAR 1. It exhibited high stability after 15 days incubation in rat serum at 37°C, along with potent and selective cytotoxicity against HER2^+^ breast cancer cells [24]. While compared with trastuzumab ADC, this anti-HER2 Fab-drug conjugate showed lower potency in cancer cells with low HER2 expression, potentially due to its reduced affinity and half-life, although further *in vivo* investigations are warranted.

As mentioned above, Fab offers advantages as a drug conjugation format. However, Fab-drug conjugates exhibit high dose dependency stemming from suboptimal internalization and pharmacokinetics. The safety implications of maximum tolerated dose cannot be disregarded, which could limit the applicability of Fab-drug conjugates to some extent. Liu et al. found that anti-CD20 Fab-vcMMAE (DAR1) had a half-life of only 2.06 h, much lower than its IgG counterpart (67.72 h). To improve therapeutic efficacy, high dose Fab-vcMMAE (20 mg/kg) was required for sufficient antitumor activity *in vivo* [25]. They further fused a mCH3mut scaffold to the Fab-vcMMAE, which increased its half-life. Nevertheless, the improvement of pharmacokinetics did not enhance anticipated antitumor potential, likely due to lower tumor accumulation. A novel anti-Trop2 Fab-DOX conjugate exhibited high nM potency and moderate efficacy when administered at a dose of 6 mg/kg every 2 days [26]. Meanwhile, it exerted high tumor inhibition rate in pancreatic xenograft mice and no animal death occurred, while DOX treatment caused three deaths, providing evidence for the safety of Fab-drug conjugates.

### Single-chain variable fragment (scFv)

The scFv comprises heavy- and light-chain variable region of antibody connected by a short linker peptide consisting of 15–20 amino acids [27]. Due to their small size, scFvs can penetrate tumors more efficiently than mAbs for solid tumors [20]. The scFv-drug conjugates (SDCs) have gained considerable attention for targeted therapy.

To enhance the safety of SDCs, an innovative application is targeted photodynamic therapy, separating the cytotoxicity from tumor targeting and requiring photosensitizer activation by light, conferring low side effects [28]. Deonarain et al. pioneered scFv-photosensitizer SDCs, demonstrating potent cytotoxicity against tumor cells *in vitro* and tumor eradication at 4 mg/kg in a HER2^+^ tumor model [29]. However, the development of photodynamic SDCs has been impeded by its intricate nature. Ongoing researches showed that optimal linkers and payloads are required to match scFv properties like aggregation, binding, and stability [30]. An early SDC comprised scFv-DOX at 1:20 ratio via polyaldodextraven (PAD) linkage, exhibiting potent cytotoxicity toward MK+ cells and inhibition of tumor growth *in vivo* [31]. Another method to prepare SDCs is fusion protein through gene editing. SNAP-tag specifically and rapidly conjugates benzylguanine (BG)-modified drugs in a defined 1:1 stoichiometry [32]. Woitok et al. conjugated BG-modified auristin F with SNAP-tagged anti-EGFR and anti-HER2 scFvs, demonstrating efficient cytotoxicity in EGRF/HER2-expressing cells [33]. Additionally, Nicolas and co-workers developed scFv-MMAE or scFv-MMAF conjugates using second generation maleimide, exhibiting selective cytotoxicity [34]. However, SDCs with DAR 1 exhibit lower potency compared with previously reported ADCs with DAR 4. Except for scFv formats, minibodies (scFv-CH3 dimers) have also been developed as targeted vectors. Recently, two different Ganglioside GD2-binding minibodies and scFv fragments identical to those of dinutuximab were site-specifically conjugated to MMAE or MMAF using thiol-maleimide chemistry with DAR of 2 and 1, respectively [35]. Both formats elicited selective cytotoxic and cytostatic effects exclusively in GD2-positive neuroblastoma and melanoma cell lines, with IC_50_ values in the nanomolar range. The cytotoxic effects and antigen binding of minibody-based SDCs were more pronounced compared with scFv-based SDCs due to their higher DAR and stronger bivalent interaction with the antigen.

Robust strategies to design highly potent SDCs focus on optimizing their affinity for binding antigens and internalization, as well as extending their half-life. In terms of internalization, phage display technology was employed to generate a repertoire of scFv and scFv-Fc fragments, which were subsequently assessed for their kinetics of internalization [36]. The study found that there was a positive correlation between the relative rates and levels of internalization of scFv-phage antibodies with their scFv and scFv-Fc forms. One example is a 5 T4-targeting scFv-Fc- auristatin F SDC with high DAR (DAR 10–12) that showed high affinity for the 5 T4 antigen [37]. It was selectively bound to and internalized into 5 T4-expressing tumor cells, and potent cytotoxicity was demonstrated for a diverse panel of solid tumor cell lines. Additionally, high doses were not deemed necessary, a single IV dose as low as 1 mg/kg resulted in complete regression of tumors and led to the emergence of tumor-free survivors in the A431 cervical cancer model. Regarding the aspect of SDC s affinity, a fascinating study conducted by Wang et al. introduced a novel strategy to exploit the macro-pinocytosis observed in pancreatic cancer [38]. They recombinantly fused an anti-EGFR scFv with domain III of human serum albumin and conjugated with lidamycin. Although clear specificity was not demonstrated, high potency was observed in four pancreatic cancer cell lines with IC_50_ range 15–70 pM. Wang’s team further conducted a novel SDC targeting tumor microenvironment, composing of a platelet integrin targeted scFv and MMAE [39]. *In vivo* studies confirmed that the newly SDC localized to primary tumors and metastases in a mouse xenograft model of triple negative breast cancer and four doses of 6 mg/kg resulted in a moderate tumor growth delay, demonstrating proof-of concept for tumor treatment that lack specific molecular epitopes for drug targeting. In conclusion, the application of highly potent SDCs still holds significant potential in ADC landscape and clinic translation.

### Bivalent antibody-derived fragments

Bivalent antibody-derived fragments, such as F(ab′)2, small immune proteins (SIPs), minibodies, and diabodies, enable simultaneous targeting of multiple epitopes of antigen, presenting advantages for drug conjugate design compared with traditional monoclonal antibodies. For instance, an anti-VEGFR2 F(ab′)2 which was conjugated with the mitochondria targeted antioxidant peptide SS31 conferred the advantage of attenuating oxidative stress and better therapeutic response in diabetic nephropathy mice model, validating the effectiveness of bivalent antibody fragments in the development of ADCs for Diabetic nephropathy therapy [40]. Kim and co-workers engineered anti-CD30 diabodies with cysteine residues for site-specific conjugation to MMAE or F (DAR 4). Despite the faster clearance of diabody, *in vivo* potency and efficacy comparable to corresponding IgG-ADC only led to a 3-fold drop [41]. Similarly, Neri et al. conducted a F8 anti-EDA (fibronectin) SIP-drug conjugates conjugated with DM1 (DAR 2) [42]. The result showed that SIP-ADC was more efficacious than IgG-ADC at equal milligram doses. Even though the SIP-ADC accumulated into the tumor and cleared more rapidly, the 24 h uptake levels were more than 4-times higher than the IgG ADC, suggesting that the higher tumor payload had a major influence to *in vivo* efficacy. In summary, bivalent antibody-derived fragments are an innovative format that provides versatility in selecting antibody scaffolds, targeting specificities, and designing pharmacokinetic properties to ADC therapeutic potential.

## BASED ON SINGLE-DOMAIN ANTIBODIES

### Nanobody

A promising approach for next-generation targeted drug conjugates utilizes highly stable and remarkably specific nanobody (Nb) that consists of only heavy-chain variable domain, known as Nb-drug conjugations (NDCs) [43, 44]. Panikar et al. extensively discussed Nb generation through phage display screening, as well as the conjugation techniques for designing target-specific nanocarriers, particularly highlighting the potential of NDCs in cancer therapy [45]. Recent advancements demonstrate greater potential of NDCs for clinical applications.

The most notable distinction with ADC is that Nbs demonstrate remarkable plasticity, making them highly suitable for integrating diverse functional modules, increasing the clinical application potential of NDCs. Leveraging the distinctive properties of Nbs, Huang et al. devised a multifunctional NDC platform for targeted delivery of platinum (IV) prodrugs [46]. The prodrug is site-specifically conjugated to the Nbs through a C3-tag and Gd3+ is loaded into a high-affinity Gd-binding domain. Compared with cisplatin, the biparatopic anti-EGFR NDC enables efficient internalization into EGFR-positive cancer cells due to 4–5 fold higher tumor accumulation, thereby enhancing tumor-specific drug delivery while minimizing systemic distribution. Moreover, recent studies have reported Nb conjugation with immune receptor agonists. For instance, Yu et al. developed PD-L1/TLR7 dual-targeting NDCs [47], and Bolli et al. investigated the anti-macrophage mannose receptor Nb-IMDQ conjugates [48]. These studies have demonstrated that the NDCs, comprising targeted Nbs and toll-like receptor activators, can effectively achieve tumor enrichment, resulting in a significant declined tumor growth and modulation of the tumor immune microenvironment. Moreover, NDCs can also exert influence in inflammatory settings. Dexamethasone-conjugated Nbs specific to granulocytes and other myeloid cells achieved targeted delivery to inflammatory sites, resulting in mitigated weight loss in virus-infected mice [49]. In contrast, equivalent free dexamethasone had no discernible effect. Furthermore, single-domain Variable New Antigen Receptors (VNARs) derived from sharks and cartilaginous fish have been engineered into novel NDCs by conjugating with diverse agents [50, 51]. Remarkably, VNAR-Fc MMAE conjugates demonstrated remarkably sub-nanomolar potency against lymphoma [52]. Overall, NDCs demonstrate potential for targeted therapeutics for treating cancers, infections, and immune diseases.

Current NDCs research focuses on addressing clearance rates, tumor tissue permeability, and immunogenicity. To extend half-life of NDCs, a half-life extender (HLE) comprising an albumin-binding VH domain has been developed to overcome rapid renal clearance [53]. Xenaki et al. used HLE technology to investigate whether extending half-life through non-covalent albumin binding could enhance the efficacy of a HER2-targeted NDC [54]. The results demonstrated that NDCs were internalized by HER2-expressing cells, regardless of the presence of albumin. HLE fusion led to a 14.8-fold increase in serum half-life and prolonged the intratumoral accumulation of NDCs in HER2-expressing xenografts without compromising distribution. Currently, various strategies used for half-life extension of Nbs, such as PEGylation or Fc fusion, also can be applied to NDCs [55, 56].

### Fully human sdAb

To facilitate the clinical use of NDCs, it is advisable to develop fully human Nbs as a precaution against potential immunogenicity. We recently synthesized a novel type of single-domain antibody by incorporating human naive complementarity-determining regions (CDRs) into a germline IGHV framework region [57]. Due to the fully human origin, the antibody was named as fully human single-domain antibodies (UdAbs). Based on this, we developed UdAb n501 specifically targeting the oncofetal antigen 5 T4 and subsequently conjugated it with SN38 using site-specific coupling. The n501-SN38 UdADC demonstrated remarkable homogeneity and potent cytotoxicity against the BxPC-3 carcinoma cell line expressing 5 T4, with an IC_50_ value of 11.9 nM [58]. Furthermore, the UdADC exhibited superior penetration capability in both multicellular tumor spheroids and patient-derived pancreatic tumor organoids, surpassing that of conventional IgG1-based ADCs. The UdADC is well tolerated in healthy mice at high doses during a 2-week period. Notably, compared with other NDCs, UdADCs have potential to offer enhanced safety and efficacy in humans owing to their human origin [59].

In addition, human single-domain antibody can also be generated using a proprietary transgenic mouse platform Humabodies, developed by Crescendo Biologics® [60]. Nessler et al. utilized this platform to evaluate the impact of cellular internalization on NDC [56]. They developed two PSMA-targeting NDCs carrying HLE-DGN549, a low-affinity monovalent (VH1) and a high-affinity biparatopic (VH1-VH2). Interestingly, despite reduced *in vitro* potency and internalization, the VH1 NDC displayed enhanced *in vivo* efficacy compared with the VH1-VH2 NDC. The superior *in vitro* potency of the biparatopic NDC constructs is likely attributed to receptor surface clustering and rapid drug internalization. However, *in vivo*, the extravasation of NDCs from the bloodstream constitutes the rate-limiting step and governs overall tumor uptake. This may play a pivotal role in determining the efficacy.

### Heavy-chain antibody

In order to combine the high tumor penetration capabilities of sdAbs and the long serum half-life, researchers have engineered heavy-chain antibodies lacking the light chains by fusing VH domains with the Fc region of human IgG1. These heavy-chain antibodies exhibit similar pharmacokinetic properties and Fc-mediated effect functions as full-length IgG antibodies [61]. Recently, Sun et al. conjugated the cytotoxic MMAE payload to the Fc region of the VH-Fc antibody targeting Mesothelin (MSLN) with a DAR of 3.6 [62]. The VH-Fc-MMAE ADC exhibited strong killing activity against NCI-H2452 cells with EC50 of 8 nM, and exhibited *in vivo* potency, effectively suppressing tumor growth at a dosage of 2 mg/kg. However, toxicity was observed in mice at a dose of 10 mg/kg. Since the conjugation is through the Fc region, this approach is similar to traditional IgGs and may not adequately address safety concerns and manufacturing challenges. Additionally, although the molecular weight of VH-Fc is lower than intact IgG, the overall molecular weight of VH-Fc-MMAE conjugate is still quite larger than sdAbs. This relatively large size may still limit tissue penetration and impede the ADC from reaching targeted tumor cells efficiently. While the VH-Fc-MMAE ADC exhibited potent antitumor activity in mice models, further optimization is likely needed, especially regarding the linker location. Potential alternative strategies could explore site-specific conjugation to the VH region or usage of smaller antibody fragments.

### BASED ON ANTIBODY-LIKE SCAFFOLDS

Several limitations of recombinant antibody technology have prompted the emergence of non-Ig binding proteins. Early examples encompassed Affibody, Monobody (Adnectin), and designed ankyrin repeat proteins (DARPins), which were derived from fragments of streptococcal protein A, the 10th type III domain of human fibronectin, and ankyrin repeat proteins, respectively [63]. Except DARPins that can be assembled，these protein scaffolds typically range in size from 6 to 13 kDa, which is smaller than most antibody fragments. Recently, several researchers have tried to conjugate these smaller protein scaffolds with cytotoxic drugs, thereby creating small molecules that facilitate targeted delivery of anticancer therapeutics.

### Fibronectin type III (FN3) domain

Adnectins are a family of engineered proteins, comprising ~100 residues (10 kDa), designed based on the structural framework of the 10th human fibronectin type III (FN3) domain. They lack the disulfide bridge connecting β-sheets, enabling cysteine-free conjugation. The Adnectin platform is being commercialized by BMS, and BMS-986089 has been evaluated in Phase 3 clinical trial [64]. To enable targeted delivery, Shalom et al. developed an EGFR-targeting Centyrins using a highly stable FN3 domain and identified optimal cysteine conjugation sites for a maleimide-linked MMAF by high-throughput screening [65]. One of Centyrins-MMAF conjugates exhibited *in vitro* potency with an IC_50_ of 0.2 nM. Furthermore, it is demonstrated that the Centyrin scaffold exhibited high compatibility with conjugation, as 26 of 94 sites tolerate cysteine mutation to payload conjugation without compromising activity. An Adnectin targeting Glypican-3 was conjugated to a cytotoxic derivative of tubulysin, demonstrating high stability and cytotoxicity both *in vitro* and *in vivo* [66]. Weekly administration of these conjugates achieved sustained antitumor effect, while a single dose of Adnectin-drug conjugate inhibited tumor growth for 2 weeks, indicating extended half-life may not be necessary for efficacy.

### DARPins

DARPins comprise ankyrin repeat proteins (14–17 kDa), possessing a groove-like surface, efficient folding, and high solubility. The DARPin platform is commercialized by Molecular Partners [67]. DARPin scaffold is intentionally designed without internal cysteines, enabling facile introduction of a unique cysteine at any position to enable precise site-specific drug conjugation [68]. By employing site-specific conjugation, Brandl et al. systematically investigated the impact of size on the tolerability and antitumor efficacy of a series of DARPin-cytotoxin conjugates (Ec1-MMAF) that specifically target the epithelial cell adhesion molecule (EpCAM) expressed on solid tumors [69]. They found that medium-sized conjugates exhibited superior antitumor effects. Furthermore, an additional study on DARPin-drug conjugation specifically investigated on bivalent EGFR-targeting DARPin-MMAE conjugates (DAR 2) and monomeric DARPin-Fc-MMAE conjugates (DAR 4), which effectively killed EGFR-overexpressing A431 cells with sub-nanomolar potency [70]. Additionally, tumor targeted-DARPin has also been conjugated to anticancer proteins like fragmented human lactoferrin (rtHLF4), Cecropin A (CRPA), Cecropin CMV (CRPC). The new conjugates exhibited improved anticancer efficacy by over 100-folds *in vitro* and *in vivo* [71].

### Affibodies

Affibodies are a class of small (58 aa), non-Ig proteins (6–7 kDa) with a three-helix bundle structure, derived from engineered IgG-binding domain of bacterial protein A. They are currently being developed by Affibody AB, a Swedish enterprise, and undergoing Phase 2 clinical trials [68]. Similarly, the cysteine in the affibody enables precise payload conjugation. Current research focuses on HER2-targeting affibody drug conjugates (AffDC). Otlewski et al. synthesized two AffDCs by conjugating MMAE with monomeric ZHER2:2891 and dimeric ZHER2:4, exhibiting low nM potencies against HER2^+^ cells [72]. To extend the half-life of AffDCs, Xu et al. fused dimeric ZHER2:2891 to an albumin binding domain (ABD) for maytansine conjugation [73]. These AffDCs showed 270–470 pM potency *in vitro* and significantly suppressed tumor growth *in vivo*, although requiring high dose of 8.5 mg/kg for five times per week. A latest study synthesized a nanoagent by employing molecular self-assembly of an amphiphilic conjugate consisting of ZHER2:342-Cys and auristatin E derivative. The new AffDC exhibited potent tumor growth inhibition in SKOV-3 and BT474 tumor models, requiring only a dosage of 1 mg/kg once every 5 days for a total of five treatments [74]. Beyond HER2, Rinne et al. developed a HER3-targeting AffDC, ZHER3-ABD-mcDM1, which bound BxPC-3 cells with 0.2 nM affinity and demonstrated potent cytotoxicity with an IC_50_ value of 7 nM [75].

## PEPTIDES AND MOLECULES

The utilization of polypeptide-coupled drugs (PDC) facilitates the selective accumulation of cytotoxic payloads within tumor cells. The PDCs topic of designing of peptide conjugates with small molecules and their clinical translation potential has been extensively covered by He et al. [76] and Melnyk et al. [77]. There are only two PDCs currently approved by the FDA for clinical cancer treatment: Melflufen and177Lu-dotatate [78]. Here we present two concise selections of representative and recent polypeptide drug conjugations: pentarins and bicyclic peptides. Pentarins and bicyclic peptides, falling within the lower range of peptide scale (2–5 kDa), are noteworthy for their drug conjugates that have reached an advanced clinical stage [77]. The pentarins drug conjugate PEN-221 incorporates a derivative of the somatostatin analog Tyr3-octreotate and DM1. Whalen et al. reported the properties of PEN-221, which exhibits a high binding affinity of 51 pM with Somatostatin Receptor 2 (SSTR 2) ，demonstrates rapid internalization of the receptor and results in significant regression of tumors in multiple SCLC SSTR2-expressing human xenograft models [79]. Bicycle-toxin conjugates (BTC) represent a novel form, wherein the phage-displayable bicyclic peptide technology (referred to as ‘bicycles’) was discovered and developed by Heinis et al. [80]. The commercialization of BTC is currently being pursued by Bicycle Therapeutics Ltd. Many BTC are in early clinical development, and BT5528 contains a bicyclic peptide that targets the tumor antigen ephrin A2 receptor (EphA2) by connecting MMAE via cleavable linkers [81]. Weekly administration of a dosage of 0.5 mg/kg resulted in tumor regressions, demonstrating efficacy even for tumors as large as 1000 mm^3^ at doses of 3 mg/kg. An ongoing Phase I/II study is being conducted in patients with relapsed advanced solid tumors expressing EphA2.

The smallest format of drug conjugate would be those containing a small chemical moiety as the targeting ligand. The selection of small molecule-drug conjugate (SMDC) ligands is a difficulty in research and development [82]. Endocyte has been innovating and developing SMDCs for a long time. Recently, Zana et al. developed a novel OncoFAP-MMAE SMDCs, which designed to be selectively cleaved by Fibroblast activation protein (FAP) in the tumor microenvironment [83]. The novel SMDC was electively delivered more than 10% injected dose per gram of MMAE to FAP-positive tumors, with a tumor-to-kidney ratio of 16:1 at 24 h post-injection, which was identified as the best performing SMDC, which has now been prioritized for further clinical development.

## DISCUSSION AND PERSPECTIVES

While IgG-drug conjugates represent a mature technology refined over decades, ADC research is progressing into an innovative transitional phase as elaborated herein. Multiple novel smaller antibody formats are emerging as substitutes for conventional IgG scaffolds, including Fabs, scFvs, and sdAbs. Such innovative smaller format antibody-drug conjugates (FDCs) are poised to overcome limitations of traditional IgG-based constructs and provide an alliterative promising antitumor therapeutics.

**Figure 1:**
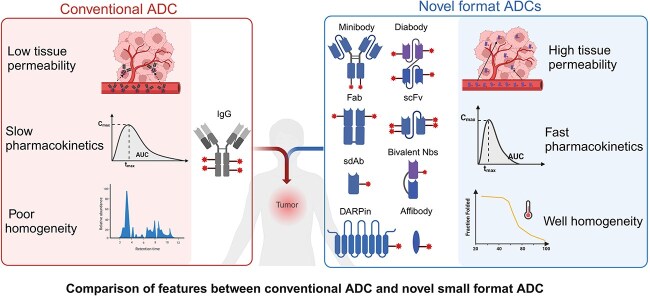
Conventional ADCs composing of IgG suffer from issues such as poor tissue permeability and suboptimal efficacy, attributed to their large volume and poor homogeneity. While the utilization of novel recombinant antibody formats as drug conjugates offers a promising approach for more precise cancer targeting. One significant advantage of these recombinant antibody formats is their smaller size, which allows for optimal penetration into solid tumors. This characteristic enables them to reach deep-seated tumor cells that may be difficult to target with conventional therapies. Moreover, these novel format ADCs have different pharmacokinetics from conventional ADCs, manifested by faster peaking and clearance. Last, recombinant antibody formats possess enhanced physical and chemical properties, rendering their drug conjugates conferring favorable characteristics.

Owing to smaller sizes, these antibody fragments and scaffolds exhibit optimal solid tumors penetration and bind to concealed epitopes that are inefficient recognized by mAbs, which making their application as drug conjugates offering more precise cancer targeting [18, 63]. Additionally, a major challenge in generating efficacious ADC is conjugating sufficient drug payloads while avoiding heterogeneity [84]. The smaller modular nature of FDCs enables precise chemical or site-specific conjugation to improve homogeneity and stability. Although FDCs theoretically possess lower drug payload capacities compared with mAb-based ADCs, they can achieve more effective and stable accumulation in tumor sites [24, 34, 58]. These inherent advantages compensate for their payload restrictions and enhance FDCs localization and retention at tumor. Furthermore, antibody fragments can be engineered into multivalent formats with enhanced tumor-targeting and specificity by synergy targeting multiple epitopes of antigens, further contributing to improve therapeutic efficacy and safety profiles [40, 56] (Fig. 1, Table 1).

Notably, an emerging viewpoint is that moderate-affinity ADCs exhibit superior therapeutic potential over high-affinity counterparts. *In vivo* efficacy is not solely dictated by tumor uptake or *in vitro* potency, as high-affinity binding may restrict tumor penetration and distribution [56]. Tight binding impedes ADC diffusion into poorly perfused regions and concealed antigen sites. Computational models incorporating binding kinetics, physiology, target expression, and ADC properties agree with *in vitro* and *in vivo* evidence that moderate affinity enhances total tumor exposure. Our prior work with tumor spheroids substantiates moderate affinity conferring improved penetration over high affinity [58]. Fundamentally, affinity must be balanced against stringent ADC requirements including stability, solubility, tumor distribution, and uptake. Very high affinity could negatively impact pharmacokinetics, while low affinity may reduce targeting specificity. The ongoing clinical trials of FDCs will give us an overview of how we can continue to boost and improve them in the near future.

Currently, FDCs facing major challenge is faster renal clearance, often requiring high and frequent dosing [25, 85] (Table 2). Although half-life extension techniques like HLE have been employed, whether prolonged exposure definitively improves FDC efficacy or safety remains inconclusive and needs to be validated by further investigations. With continued innovation and application of FDCs, such therapeutics hold potential for improved pharmacology and safety profiles, enabling precise cancer targeting.

**Table 2:** Characteristics of smaller format drug conjugates compared with mAbs-based ADC

Formats	Advantages	Disadvantages
Bivalent fragments	Multivalent antigen targeting High tumor accumulation	Low potency *in vivo* compared with ADC with same DARs
Fabs	Enhanced homogeneity and stability Potent cytotoxicity *in vitro*	Low tumor accumulation High dose dependency Moderate efficacy *in vivo* compared with ADC with same DARs
ScFvs	Tailorable linkers and payloads Potent cytotoxicity *in vitro* Better internalization of tumor cells	Low potency *in vivo* compared with ADC with same DARs
Nbs	High stability and plasticity Conjugate with different forms of drugs High tumor accumulation and permeability Potent cytotoxicity *in vitro* Multiple humanized platforms	Frequent administration due to short half-life
Scaffolds	Potent cytotoxicity *in vitro* High compatibility with drug conjugation	Frequent administration due to short half-life
Peptides and molecules	Smaller size and precision structure Potent cytotoxicity *in vitro* Rapid internalization High tumor accumulation and permeability	Frequent administration due to short half-life

## Data Availability

No new data were generated or analyzed in support of this research.
