# Correction to: Reforming solid tumor treatment: the emerging potential of smaller format antibody-drug conjugate

**DOI:** 10.1093/abt/tbae015

**Published:** 2024-06-17

This is a correction to: Xiaojie Ma, Mingkai Wang, Tianlei Ying, Yanling Wu, Reforming solid tumor treatment: the emerging potential of smaller format antibody-drug conjugate, *Antibody Therapeutics*, Volume 7, Issue 2, April 2024, Pages 114–122, https://doi.org/10.1093/abt/tbae005.

The published article has been updated to include an Ethics and consent statement and an Animal research statement, which were inadvertently omitted during the publishing process.

